# Isolated pancreatic tuberculosis mimicking as carcinoma: a case report and review of the literature

**DOI:** 10.1186/1757-1626-3-18

**Published:** 2010-01-12

**Authors:** Sudeep Khaniya, Rabin Koirala, Vikal Chandra Shakya, Shailesh Adhikary, Rajendra Regmi, Sagar Raj Pandey, Chandra Shekhar Agrawal

**Affiliations:** 1 Department of Surgery, B. P. Koirala Institute of Health Sciences, Dharan, Nepal; 2 Department of Pathology, B. P. Koirala Institute of Health Sciences, Dharan, Nepal

## Abstract

**Introduction:**

Pancreatic tuberculosis is a rare disease even in endemic countries for tuberculosis. Here, we report a case of pancreatic tuberculosis from tuberculosis endemic zone presenting as obstructive jaundice mimicking pancreatic cancer.

**Case presentation:**

A 41-year-old male presented with features of malignant obstructive jaundice. Ultrasonography and computed tomography scan showed mass in the pancreatic head and uncinate process. He underwent a pancreatoduodencetomy. Histological examination showed typical features of tuberculosis. Antitubercular drugs were started and he remains well six months after surgery.

**Conclusion:**

Tuberculosis should be considered as a differential diagnosis to an obscure pancreatic mass in younger or middle aged patient residing in tuberculosis endemic zone.

## Introduction

Tuberculosis (TB) is a common disease in developing countries, and even in developed countries, it is becoming important, especially with the rise of acquired immunodeficiency syndrome and widespread use of immunosuppressant drugs. Pancreatic TB is considered to be extremely rare. Most cases of pancreatic TB are diagnosed only after tissue biopsy or exploratory laparotomy. Because almost all cases of pancreatic TB are effective to antituberculosis management, every effort should be made to arrive at an early diagnosis so as to avoid unnecessary interventions, including laparotomy. Here we present a case of TB in the pancreatic head and uncinate process mimicking pancreatic carcinoma in a 41 year-old male.

## Case presentation

A 41-year-old Nepalese male of Aryan origin presented with 3 months history of gradually progressive jaundice, intermittent right upper quadrant pain, and weight loss of 5 kg over a 2-month period. There was no history of cough, fever, hemoptysis or shortness of breath. He had received a BCG vaccine at childhood, but there was no prior history of tuberculosis, or family history of contact.

**Figure 1:**
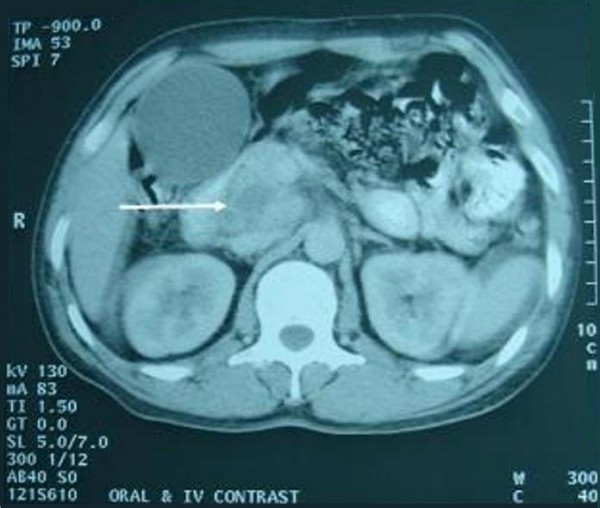
**Contrast enhanced CT abdomen showing heterogeneous lesion in the head and uncinate process of pancreas with non enhancing areas suggestive of necrosis (arrow) and distended gall bladder**.

**Figure 2:**
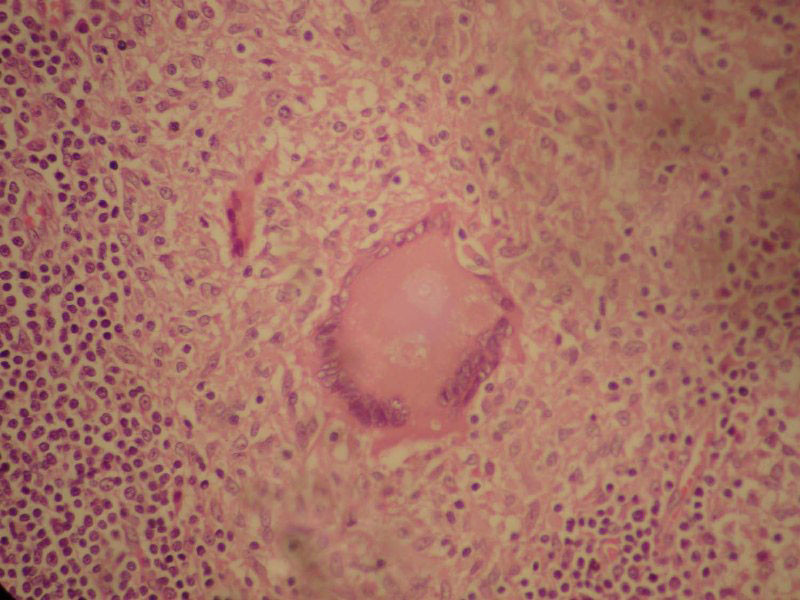
**Photomicrograph showing pancreatic parenchyma destroyed by granuloma (×400 H&E stain)**.

On examination, patient was deeply icteric with skin scratch marks all over the body. On abdominal examination, he had mild hepatomegaly and a palpable gall bladder. Initial laboratory values revealed a WBC count of 9200/mm^3 ^(90% neutrophils, 10% lymphocytes), haemoglobin 12.7 g/dL, total bilirubin 24.4 mg/dL, conjugated bilirubin18 mg/dL, ALT 96 U/L (normal 5-45 U/L), AST 161 U/L (normal 5-45 U/L), and ALP 593 U/L (normal 42-128 U/L), albumin 3.5 mg/dL and total protein 5.7 mg/dL. His random blood sugar, serum urea and creatinine will within normal limits. A chest X-ray film was normal. Abdominal ultrasound examination revealed an irregular hypoechoic lesion of 3 cm × 4.4 cm in the head and uncinate process of pancreas, and with dilation of entire bile duct system, distended gall bladder with normal pancreatic duct. Contrast enhanced CT scan showed a heterogeneous mass in the pancreatic head and uncinate process of the pancreas. (Figure 1) The gall bladder was distended along with the dilatation of entire biliary tract. Exploratory laparotomy revealed a mass of size 3×2 cm at the head and uncinate process of pancreas with areas of necrosis on cut section, 50 ml of thick pus at the retroduodenal region, dilated distal common bile duct, distended gall bladder and multiple enlarged perichodochal and peripancreatic lymph nodes. The patient underwent pancreatoduodencetomy with intraoperative diagnosis of pancreatic carcinoma. AFB staining of the pus was negative and culture was sterile. However, the histopathology from the pancreatic mass revealed necrotizing granulomatous lesion which was positive for acid fast bacilli. The histopathological picture of the enlarged nodes was of reactive lymphadenitis. (Figure 2) The patient had uneventful postoperative period. After the diagnosis of tuberculosis, antitubercular drugs were started in accordance to DOTS (Directly observed treatment, short-course), and patient is doing well at the follow up after 6 month.

## Discussion

Tuberculosis is a major public health problem in developing countries. Though TB commonly occurs in lung, primary abdominal TB is not uncommon, incidence ranging from 0.58% to 12% [1]. But contrary to belief, only 6-38% of patient with active pulmonary TB have abdominal TB [2]. TB does easily disseminate to the gastrointestinal tract, liver, spleen and mesenteric lymph nodes; however the involvement of pancreas is rare. The first report pancreatic TB mimicking cancer was reported by Auerbach in 1944. In his series of 1656 autopsies of tuberculous patients, only 14 cases had direct pancreatic involvement that may have mimicked neoplasia [3]. Since then, most of the medical literature on this rare disease is limited to case reports or small case series. There have been reported incidents in the past where extensive surgeries have been performed for high suspicion of periampullary carcinomas which later turned out to be tuberculosis of the pancreas [4]. Feng Xia et al. have summarized characteristics of pancreatic TB as follows: 1) mostly occurs in young people, especially female; 2) have a past history of TB, or come from endemic zone of active tuberculosis; 3) often present with epigastric pain, fever and weight loss; 4) ultrasound and CT scan show pancreatic mass and peripancreatic nodules, some with focal calcification [5]. The other reported presentation of pancreatic TB is as follows; obstructive jaundice, gastrointestinal bleed, acute or chronic pancreatitis, pancreatic abscess, portal venous thrombosis causing portal hypertension and even colonic perforation [6,7].

Even though, the disease occurs commonly in patients residing in endemic zones, or in those with immunosuppressant status, the diagnosis of pancreatic TB is a real challenge. The challenge is partly because of rarity of the disease itself and partly due to its insidious presentation, with nonspecific signs and symptoms or mimicking pancreatic carcinoma like in the present case. Even though the patient belonged to TB endemic zone, due to its unusual presentation we suspected of malignancy and he was managed with extensive but albeit unnecessary resection. There are other reports also in which pancreatic TB has not been diagnosed preoperatively [1,4,6]. In Saluja et al study, out of 18 patients 4 patients had pancreatic TB and all required operative resection for diagnosis [4].

The noninvasive diagnostic techniques for pancreatic TB rely mainly on ultrasonography and CT abdomen. Ultrasonography reveals focal hypoechoic lesions or cystic lesions of the pancreas [8]. Findings on CT scan include hypodense lesions and irregular borders mostly in the head of the pancreas, diffuse enlargement of the pancreas or enlarged peripancreatic lymph nodes [9]. Bile cytology or ERCP has low diagnostic yield estimated around 5% [4]. In contrast to noninvasive techniques, invasive diagnostic techniques are more reliable and definitive as tissue obtained from biopsy can be assessed for microbiological and pathologic examination. Techniques for biopsy include endoscopic US-guided biopsy, CT/US- guided percutaneous biopsy, and surgical biopsy (open or laparoscopic) [10]. The microscopic features suggestive of tuberculosis are presence of caseating granulomatous inflammation and positive stain for acid-fast bacilli. Cultures for mycobacteria take up to 6 weeks to grow and are used to confirm the diagnosis. However, it must be remembered that bacteriological confirmation may not be possible in many patients [11]. The polymerase chain reaction-based assay is a highly specific assay and may give a positive result even when special staining techniques and cultures of these tissues are negative [12].

Once the tissue diagnosis has been made, the management of TB rest on the medical treatment. The treatment of pancreatic tuberculosis comprises multi-drug anti-tuberculous chemotherapy for between 6 and 12 months. The DOTS guidelines recommend only six months of therapy even for severe forms of tuberculosis [13]. Response to therapy is predictable and complete. Longer duration of treatment results in higher costs and exposes patients to more side effects.

## Conclusion

Pancreatic TB is a rare disease requiring high index of suspicion for diagnosis. Unfortunately, in most cases the diagnosis of pancreatic TB is made only after exploratory laparotomy, as in the present case. Therefore the diagnosis of pancreatic TB should be considered in the context of a mass in the head/uncinate process of the pancreas in younger patients from endemic TB zone or in the immunocompromised patients, and vigorous attempts should be made to obtain preoperative microbiological and/or histological diagnosis.

## Abbreviations

ALP: alkaline phosphatase; ALT: alanine aminotransferase; AST: aspartate aminotransferase; CT: computed tomography; DOTS: directly observed treatment shortcourse; ERCP: endoscoic retrograde cholangiopancreatography; TB: tuberculosis; US: ultrasonography; WBC: white blood count.

## Consent

Written informed consent was obtained from the patient for publication of this case report and accompanying images. A copy of the written consent is available for review by the Editor-in-Chief of this journal.

## Competing interests

The authors declare that they have no competing interests.

## Authors' contributions

SK and RK made substantial contributions to concept and design of the article. VCS, RR and SRP were involved in the acquisition of materials. CSA and SA contributed significantly in the critical revision and drafting of the manuscript. All authors read and approved the final version of the manuscript.
